# A Rare Incidence of Neonatal Button Battery Ingestion: A Case of Child Abuse and Neglect

**DOI:** 10.3390/children9111682

**Published:** 2022-11-02

**Authors:** Ahmad Zaker M Almagribi

**Affiliations:** Department of Surgery, College of Medicine, Najran University, Najran P.O. Box 1988, Saudi Arabia; azalmagribi@nu.edu.sa

**Keywords:** foreign body ingestion, neonatal, child abuse, oesophagoscopy, Saudi Arabia

## Abstract

Foreign body (FB) ingestion is not uncommon, especially when the child beings coordination of the hands and mouth from 6 months to 5 years of age. However, FB ingestion in the neonatal period is extremely rare. We present a one-month-old baby with button battery ingestion to report the unusual age of presentation, unusual clinical findings, and child abuse. A radiopaque, spherical FB was visible in the upper chest on the chest X-ray. After performing an oesophagoscopy, the battery was removed, and the patient was put on a plan of anti-GERD medications and gradual nasogastric tube feeding. The patient was coping well under the supervision of the healthcare professional. However, the patient’s father decided to take the patient home against medical advice, and since then, no follow-up has been conducted by the patient’s guardians. In conclusion, neonatal foreign body ingestion is rare, and early detection and management can save neonates’ lives. Saudi Arabia’s national child protection teams, working under the National Family Safety Program, should strictly implement approved programs to prevent child abuse and teach positive parenting skills.

## 1. Introduction

Foreign body (FB) ingestion is not uncommon, primarily when the child begins coordination of the hands and mouth from 6 months to 5 years [1]. The two main causes of the occurrence of FB ingestion are curiosity and a predisposition for oral exploration [2]. However, FB ingestion in the neonatal period is extremely rare [2,3]. Children account for 80 percent of all reported cases of FB ingestion. The coin is the most common FB, followed by the button battery, which can be lethal and result in devastating complications. Elder siblings are mainly responsible for placing FBs in newborns’ and infants’ mouths. Between 80% and 90% of FBs brought in for medical assessment can pass freely through the digestive system [4]. Ten to twenty percent require endoscopic removal, and approximately one percent require surgical intervention. When parents provide a detailed account of their child’s FB consumption, an accurate diagnosis can be made quickly. Typical diagnostic methods include lateral, anteroposterior (AP), and neck X-rays. Perforations and strictures can develop from oesophageal FB, and the erosion of neighbouring vital tissues, including the trachea and aorta is also a risk [5].

Every child with inexplicable vomiting or respiratory symptoms should be suspected of foreign body ingestion, regardless of age or whether parents/relatives are aware. Because the interval between foreign body ingestion and the onset of symptoms in children can be unpredictable, urgent therapy is indicated as soon as the patient arrives [6].

Here, we present a one-month-old baby with button battery ingestion to report the unusual age of presentation and discuss considerations in management and risk of abuse. An unusual child presentation should always raise the alarm for child negligence and abuse.

## 2. Case Report

**Figure 1:**
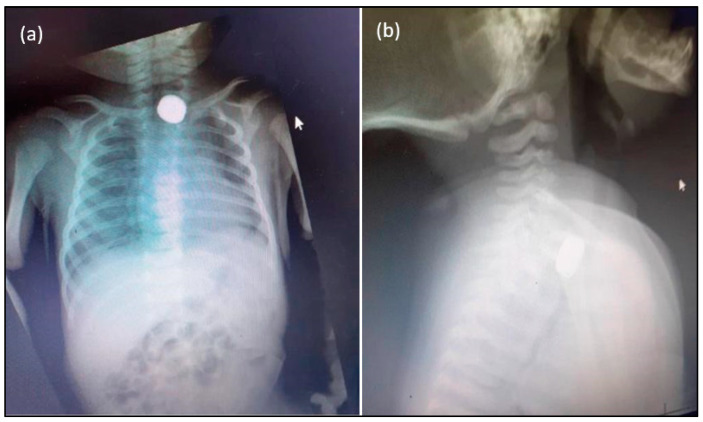
Posteroanterior (**a**) and lateral X-ray (**b**) views show metallic circular FB (battery) in the upper oesophagus.

A one-month-old baby was presented to the emergency department with complaints of choking and difficulty in feeding for ten days. History by the mother stated that the baby was fine till her elder brother introduced a foreign body into her mouth. The mother was not sure about the nature of the foreign body. The neonate was choking with shortness of breath, but the child calmed down later. The family had since travelled for a week. The mother noticed that her breathing difficulties and poor feeding persisted. They sought medical opinion in a private clinic, where the child was given nebulization and antibiotics and then discharged. Vomiting and poor feeding had not improved. They visited the emergency department of Najran maternity hospital, ten days after the foreign body ingestion event, and were referred to the otolaryngology team. The mother gave the history of another sibling’s death due to post-tonsillectomy severe pneumonitis two weeks before this presentation. The neonate’s at birth data: Normal term spontaneous vaginal delivery. Familial conditions: Middle-socioeconomic status. The details of the battery: Diameter: 5.8 mm × 2.7 mm; Voltage: 1.55v; Type: Silver-oxide. The vital signs were recorded before the chest X-ray. Vital signs were stable with equal bilateral air entry; O_2_: 98%; Temperature: 37 °C; Blood pressure: 100/59 mmHg; Pulse:159 beats/min; RR: 40 bpm; Wt: 3 kg. Clinical examination data: CNS: Conscious alert; ENT: normal examination; Chest: equal bilateral air entry with normal heart sounds; Abdomen: soft and lax. The chest X-ray showed a radiopaque, rounded foreign body in the upper chest, mostly suggestive of a button battery, as shown in Figure 1. After the child abuse committee was notified, consent was obtained to remove the battery under general anaesthesia.

**Figure 2:**
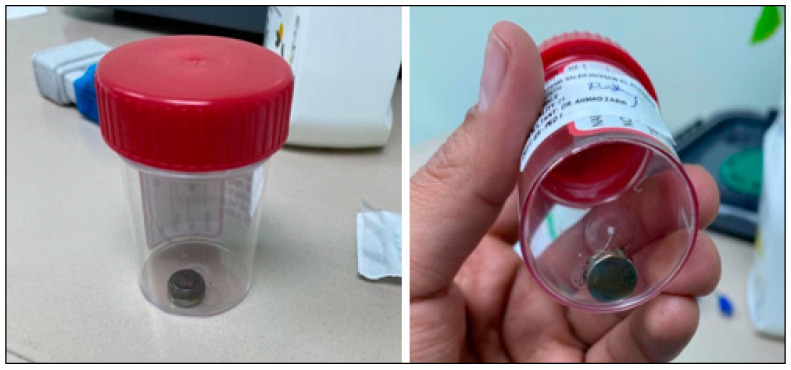
Foreign body (Button battery Silver-oxide type) immediately after removal.

A rigid oesophagoscopy (age-related: 0–3 months: Size 3) was started, and a rigid tube was introduced down the oesophagus while the patient was under general anaesthesia. Initially, the surgeon had trouble finding and removing the foreign body. A Magill baby forceps was eventually inserted via the tube to get a hold of the battery and retrieve it from the oesophagus (Figure 2). A grade two mucosal injury (niche) was noticed at the location of the battery stuck in the oesophagus, and a nasogastric tube was inserted under the vision and confirmed at the stomach intraoperatively to facilitate healing and feeding. The patient was shifted to the neonatal intensive care unit for observation for 24 h and put on a plan of anti-GERD medications and gradual nasogastric tube feeding. The patient was coping well and recovering under the healthcare professional’s supervision. Unfortunately, the father decided to take the child home against medical advice, and since then, no follow-up or other visit to our hospital has been found in the patient’s medical records. According to hospital procedure, patients who leave against medical advice should not receive treatment or follow-up, but a social worker contacted the father via hospital phone to check in on the child and he responded that there was no need for follow-up because the child was doing well.

## 3. Discussion

Although neonatal FB ingestion is rare, it can be fatal or carry lifelong morbidity for the patient [2,5]. The literature, however, supports a younger age threshold for foreign body ingestion, between 5 and 12 months; the usual age is from 6 months to 5 years [1]. A few cases of neonatal FB consumption have been mentioned in the literature. In 2000, Al-Odaidan et al. reported a 20-day-old child with a nail-like oesophageal foreign body, prompting concerns about child abuse [1]. Dian Adi Syahputra et al. described a 28-day-old newborn who swallowed his mother’s pendant, which was subsequently removed with no difficulties using a flexible endoscope [7]. A 7-day-old newborn with a nail impacted into the oropharynx introduced by an older brother and removed using a direct laryngoscope was described by Kazi et al., [5].

Furthermore, two cases reported battery ingestion into the oesophagus for more than ten days and were removed with an endoscope. The edematous and inflammatory oesophageal mucosa showed no signs of blackening, charring, or perforation [8,9]. History of foreign body ingestion conveyed by attendants and chest and cervical X-rays are the main diagnostic elements for such cases [10]. Although this case of a 20- day-old female neonate with battery ingestion in the oesophagus for ten days was successfully removed without major complications, the presence of a button battery for ten days or more without perforation or local oesophagal complication was not usual. Still, it could be explained either by the battery charge being empty or that neonates have a lower risk for penetration by battery for an unclear reason as supported by this presentation and two case reports in the literature [8,9]. In 2004, Tasneem et al. reported two cases, both of which were less than 72 h old; one had an endotracheal tube, while the other had a finger ring stuck in their oesophagus, which was retrieved using a pediatric bronchoscope with optical forceps [2]. Hernot et al. reported the case of a 2-month-old male child with an impacted metallic FB (zipper) directly below the cricopharynx. The patient’s symptoms improved once the zipper was removed by oesophagoscopy [6].

Skrzypczak et al., 2020, reported a case of button battery ingestion by a 3-year-old boy, who presented with more severe complications such as tracheo-esophageal fistula and increasing respiratory failure [11]. A tracheotomy was performed, and a bilateral bronchopulmonary tube was inserted to help with the patient’s respiratory failure. On day 25, an effort was made to reconstruct the oesophagus to the trachea. The surgical team removed the cartilage lobe from the patient’s left ear, switched to extracorporeal circulation, and then performed an end-to-end anastomosis of the patient’s oesophagus and cartilage reconstruction of the tracheal loss. The communication between the trachea and the oesophagus was identified on day 45. Following the creation of an esophagostomy, botulinum toxin was injected into the patient’s salivary glands. An examination showed that the anastomosis was indeed very secure. On the 103rd day of his hospitalization, the patient, whose overall health had improved, was discharged [11].

The prognosis is extremely grim for injuries to the trachea, bronchi, or oesophagus. Most foreign bodies should only be taken out of the gastrointestinal tract if they cause symptoms or have not left after the right amount of time has passed. Batteries and sharp objects, on the other hand, should always be removed through an endoscope within 24 h. But where the battery is located is very important [12]. Some authors say that endoscopic intervention is important when the battery is in the oesophagus, but when there are no symptoms, the battery is in the stomach and should be treated in a more conservative way [13]. Most of the time, it is not clear how to treat tracheoesophageal fistula, and it can be controversial, especially in children [12,14,15]. It is possible to treat the patient without surgery and hope that the fistula will close on its own [15]. If conservative treatment does not work, the most important decision is whether or not to undergo surgery. It is important to decide when the patient can have the surgery.

Another atypical case of prolonged (3 months) oesophagal button battery impaction in a 15-month-old was reported by Marshalla and Williamson, 2016 [16]. In this case, the patient had been evaluated by her primary care physician three months prior for a sudden refusal to eat solid foods. Although the patient had been tolerating solid food before the age of 12 months, this was due to her not being ready for solid food advancement at the time. Three months later, the patient still refused to eat solid foods; any attempt resulted in regurgitation. This prompted the referral to gastroenterology. Reports say a chest X-ray at the clinic revealed a foreign object in the middle of the oesophagus, prompting a referral to the ER where otolaryngology could assess and potentially remove it. The lesson to be learned from this case is that primary care physicians need to be made more aware of this diagnosis. A high index of suspicion for the diagnosis of button battery ingestion is warranted in children presenting with symptoms such as coughing, vomiting, gagging, refusal to eat, regurgitation of food, or chest pain because of the potentially fatal nature of button battery ingestion in children [16].

To differentiate an opaque, circular, foreign body from a coin on an anterior-posterior X-ray, zoom in and look for a double ring or halo sign. A close examination of imaging is required to rapidly arrive at the right diagnosis. The negative or narrower part of the battery can help clinicians determine where the most serious tissue injury may occur and what potential complications in the patient must be considered [17].

Bhosale et al., 2016 [18] reported a male neonate who was 2 months old and succumbed to sepsis and pneumonitis on day 15 button battery ingestion. The infant was suffering from a fever and respiratory distress that had been going on for 5 days when he was brought in. Following investigation, a round, radiopaque foreign body was discovered in the patient’s upper oesophagus. The child was brought to our facility for the removal of the coins after it was determined that they had been swallowed by the child. Following a thorough examination of the X-rays, we formed the hypothesis that the patient had consumed button batteries. Under the influence of general endotracheal anaesthesia, an emergency rigid esophagoscopy and removal of the foreign body were carried out. With some effort and the use of optical forceps, a corroded button battery that measured 4 mm wide was extracted. After the operation, the child was taken care of while attached to a ventilator. He was provided with care that was supportive. On day 10, a check scopy using an ultrathin and flexible oesophagoscope revealed that the patient had developed a tracheoesophageal fistula. The child had a procedure to defunction their gastrostomy and a feeding jejunostomy, but two days later the infant passed away from sepsis caused by mediastinitis and recurrent pneumonitis. The ingestion and impaction of a button battery by an infant younger than 2 months is extremely uncommon and has not been reported in the relevant literature [18].

There is an increased need to educate parents about the health hazards of battery ingestion and target primary prevention because the management of children with button battery ingestion is a costly affair associated with unpredictability and high morbidity. It is important for those working in health care to be familiar with the various ways in which children can present after ingesting batteries. One must always be on high alert, but this is especially important when dealing with infants exhibiting nonspecific respiratory symptoms and having an early suspicion of button battery ingestion [19]. It is of the utmost significance, in particular in situations in which the child does not respond to treatment for respiratory infections. To reduce the likelihood of complications and to improve the likelihood of a positive outcome, it is of the utmost importance to take a multidisciplinary team approach, make an early referral to a pediatric/ENT surgeon, and provide care that is both swift and synchronized [20,21].

Though many of these cases are caused by elder siblings inserting FB playfully into the mouth, some cases are homicidal, as reported by Singh et al., of a 12-day-old neonate—the third female child in a family of lower socioeconomic status. The grandmother had most likely placed a button battery in the child’s mouth and kept her at home for 10 days with the assumption that she would die due to it [8]. Another rare case was of oesophagal obstruction caused due to foreign bodies (betel nut pieces) forcefully inserted in a 6-day-old female neonate by her grandfather with homicidal intention [3].

In a recent retrospective descriptive survey, the pediatric emergency department of a tertiary hospital in Lisbon, Portugal, investigated 35 occurrences of battery ingestion in children requiring endoscopic removal between January 2011 and December 2020 [22]. The study revealed that the severity of endoscopic lesions was observed to be significantly correlated with exposure duration, younger age, or battery size. Despite the existence of an emergency pediatric endoscopy team, the study found a significant increase in hospitalizations owing to battery ingestions and accompanying problems. Patients with oesophagal batteries experienced more severe mucosal damage and had lower short/long-term results. Although the existence of gastric batteries did not rule out oesophagal damage, children with gastric batteries had milder lesions.

For post-removal mitigation, Anfang et al. [23] recommended irrigating the affected area with 50–150 mL of 0.25% sterile acetic acid to neutralize the highly alkaline substrate and slow the course of damage. Jatana et al. used this method in their series of six pediatric patients [24]. After irrigation, the mucosal look improved in all reported cases, and no strictures or perforations developed. This is because liquefactive necrosis is stopped and the pH is restored to a physiological range almost instantly.

Moreover, neonates suffering from foreign body ingestion should always raise the question about the possibility of negligence or the caretaker and neonate relationship. Battery ingestion by a neonate, leaving the hospital against medical advice, and a history of a child’s death in the same family, all raise the possibility of child negligence or abuse. Such cases should necessitate the protection of the child from further sequelae. We support the suggestion to strengthen further the child National Family Safety Program (NFSP) to estimate workup and prevent child abuse in Saudi Arabia. The hospital-based child protection teams (CPTs) project of the National Family Support Program (NFSP) was given approval by the Saudi National Health Council (NHC) in 2008, and since then, 39 CPTs have been formed in major hospitals across all 13 provinces of Saudi Arabia [25]. The CPTs are governed by the National Health Council, and the NFSP is responsible for overseeing their operations and providing free training and consultations to all members of the CPT. Each CPT is comprised of a core multidisciplinary team (consisting of psychologists, paediatricians, and social workers), as well as ad hoc individuals (consisting of nurses. Surgeons, legal service providers, and others) [26,27]. In the present case. the treating physician requested that the child abuse committee be involved, but there was no clear strategy from the committee member. We are reporting this case for this and other reasons because we believe that strict reporting laws for situations of child abuse and neglect should be implemented. In some cultures, this may be seen as humiliating for the guardians.

In conclusion, neonatal foreign body ingestion is rare, and early detection and management can save neonates’ lives. Also, parents must take utmost care to ensure that children are not left unsupervised, especially in the company of their siblings, and thus avert such mishaps. The national system should strictly implement the approved programs to save children from abuse and neglect.

## Data Availability

All the data has been included in the case report.
